# Dental pulp stem cells as a multifaceted tool for bioengineering and the regeneration of craniomaxillofacial tissues

**DOI:** 10.3389/fphys.2015.00289

**Published:** 2015-10-16

**Authors:** Maitane Aurrekoetxea, Patricia Garcia-Gallastegui, Igor Irastorza, Jon Luzuriaga, Verónica Uribe-Etxebarria, Fernando Unda, Gaskon Ibarretxe

**Affiliations:** Department of Cell Biology and Histology, Faculty of Medicine and Dentistry, University of the Basque CountryLeioa, Spain

**Keywords:** DPSC, differentiation, tooth, bone, salivary gland, nerve, cell therapy

## Abstract

Dental pulp stem cells, or DPSC, are neural crest-derived cells with an outstanding capacity to differentiate along multiple cell lineages of interest for cell therapy. In particular, highly efficient osteo/dentinogenic differentiation of DPSC can be achieved using simple *in vitro* protocols, making these cells a very attractive and promising tool for the future treatment of dental and periodontal diseases. Among craniomaxillofacial organs, the tooth and salivary gland are two such cases in which complete regeneration by tissue engineering using DPSC appears to be possible, as research over the last decade has made substantial progress in experimental models of partial or total regeneration of both organs, by cell recombination technology. Moreover, DPSC seem to be a particularly good choice for the regeneration of nerve tissues, including injured or transected cranial nerves. In this context, the oral cavity appears to be an excellent testing ground for new regenerative therapies using DPSC. However, many issues and challenges need yet to be addressed before these cells can be employed in clinical therapy. In this review, we point out some important aspects on the biology of DPSC with regard to their use for the reconstruction of different craniomaxillofacial tissues and organs, with special emphasis on cranial bones, nerves, teeth, and salivary glands. We suggest new ideas and strategies to fully exploit the capacities of DPSC for bioengineering of the aforementioned tissues.

## Introduction: DPSC and tissue engineering of the oral cavity

The oral cavity is a complex multi-organic structure. Because oral tissues and organs are functionally connected at many levels, irreversible damage to any of them is likely to eventually affect the others, causing extensive malfunction. Tooth decay, periodontal disease, alveolar bone resorption, orthodontic problems, orofacial neuropathic pain, and impaired salivary gland function are conditions that seriously affect oral health of a large part of the world population. Owing to their functional connectivity, once damage is diagnosed to one organ of the oral cavity it is important to intervene rapidly and efficiently, to repair or replace the injured or lost tissues, to avoid severe degradation of oral health.

Synthetic replacement materials and prostheses (fillings, bridges, implants, etc.) have traditionally been the treatment of choice to treat dental decay. However, all functions of the original biological tooth are not fully restored by this kind of replacement therapies. Other organs of the oral cavity (e.g., nerves, salivary glands) are simply not amenable to mechanical substitution approaches. Thus, tissue engineering represents a new collection of treatment options for the complete biological regeneration of craniomaxillofacial tissues and organs. The development of this field requires three essential components: (i) stem cells, (ii) biomaterial scaffolds, and (iii) stimulating factors or inductive signals. Tissue engineering is now fully considered as an alternative to the conventional treatments for dental injury and disease, offering substantial advantages over traditional dental restoration techniques (Nör, 2006; Wang et al., 2012).

**Figure 1:**
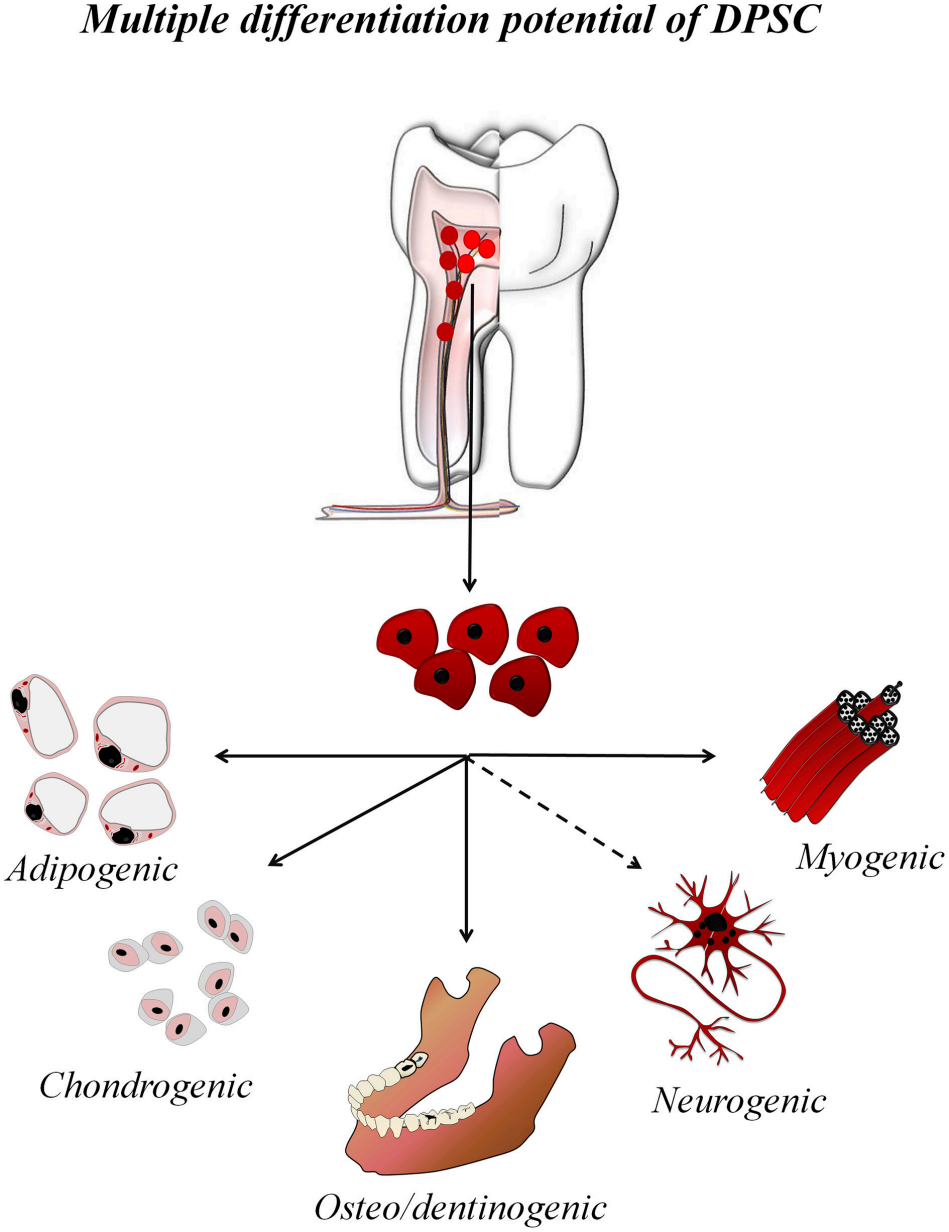
**Multiple differentiation potential of DPSC**. DPSC are isolated from the adult dental pulp and can be *in vitro* expanded and differentiated to multiple cell lineages, including all connective tissue-lineages and some neural cell lineages.

Stem cells are the cornerstone of regenerative cell therapy. An enormous variety of multipotent stem cells have been isolated and studied from different human tissues, such as the bone marrow (Ding and Morrison, 2013), adipose tissue (Kapur et al., 2015), skin (Blanpain and Fuchs, 2009), and the umbilical cord (Yan et al., 2013; Kalaszczynska and Ferdin, 2015). Among them, mesenchymal stem cells (MSC) are the most promising for clinical purposes (Rastegar et al., 2010; Ménard and Tarte, 2013). In the oral cavity, adult tooth tissues also contain different active populations of stem cells with mesenchymal phenotype (Huang et al., 2009). Unlike other types of MSC, dental stem cells originate from the neural crest (Janebodin et al., 2011) and are lineage-related with peripheral nerve glial progenitor cells (Kaukua et al., 2014), which places them in a privileged position to mediate regeneration of both connective and nerve tissues (Ibarretxe et al., 2012; Martens et al., 2013). Several types of human dental stem cells have been identified. Among them; DFPC, Dental Follicle progenitor cells; PDLSC, Periodontal ligament stem cell; SCAP, Stem cells from apical papilla; SHED, Stem cells from primary exfoliated deciduous teeth; DPSC, Dental pulp stem cells, which are without a doubt the most used for research purposes (Figure 1). Although it still remains a considerable challenge to obtain all the different types of neuronal and glial cells from DPSC, their differentiation to specialized connective tissue cells has proven to be relatively simple. Efficient protocols for obtaining adipocytes, chondroblasts and osteo/odontoblasts are firmly established in the literature (Gronthos et al., 2002; Huang et al., 2009; Kawashima, 2012).

In this review, we summarize the current understanding of DPSC and their capacity to regenerate injured orofacial tissues, with special focus on the teeth and associated periodontal tissues, peripheral cranial nerves, and the salivary glands. We present the different strategies used in experimental restorative models with potential applications in dentistry and oral medicine, together with the different scaffold biomaterials and stimulating factor combinations employed to elicit an optimal cellular response required to regenerate damaged tissues. We critically comment on some crucial aspects to be considered for bioengineering and the regeneration of each of these tissues, based on our own research experience.

## DPSC for bone regeneration

Regeneration of maxillary and mandibular bone is fundamental in the field of implantology (Lee et al., 2014). Osteogenic differentiation of DPSC is easily induced *in vitro* by adding ascorbic acid (Asc), dexamethasone (Dexa), and β-glycerophosphate (β-gly) to the culture medium, along with fetal bovine serum (Laino et al., 2005; Langenbach and Handschel, 2013). Asc is an essential enzyme cofactor to generate pro-collagen (Vater et al., 2011), which is necessary for the correct synthesis of Collagen type I, the main organic component of the bone matrix. Dexa is required for osteoblast differentiation, working as Runx2 inductor by activating WNT/β-catenin signaling in MSC (Hamidouche et al., 2008). Runx2 transcription factor expression is fundamental in driving cellular commitment to osteogenic lineages. Finally, β-gly is a donor of inorganic phosphate, which is essential for creating hydroxyapatite mineral, and as a signaling molecule to induce DPSC osteo-differentiation (Foster et al., 2006; Fatherazi et al., 2009; Tada et al., 2011).

**Figure 2:**
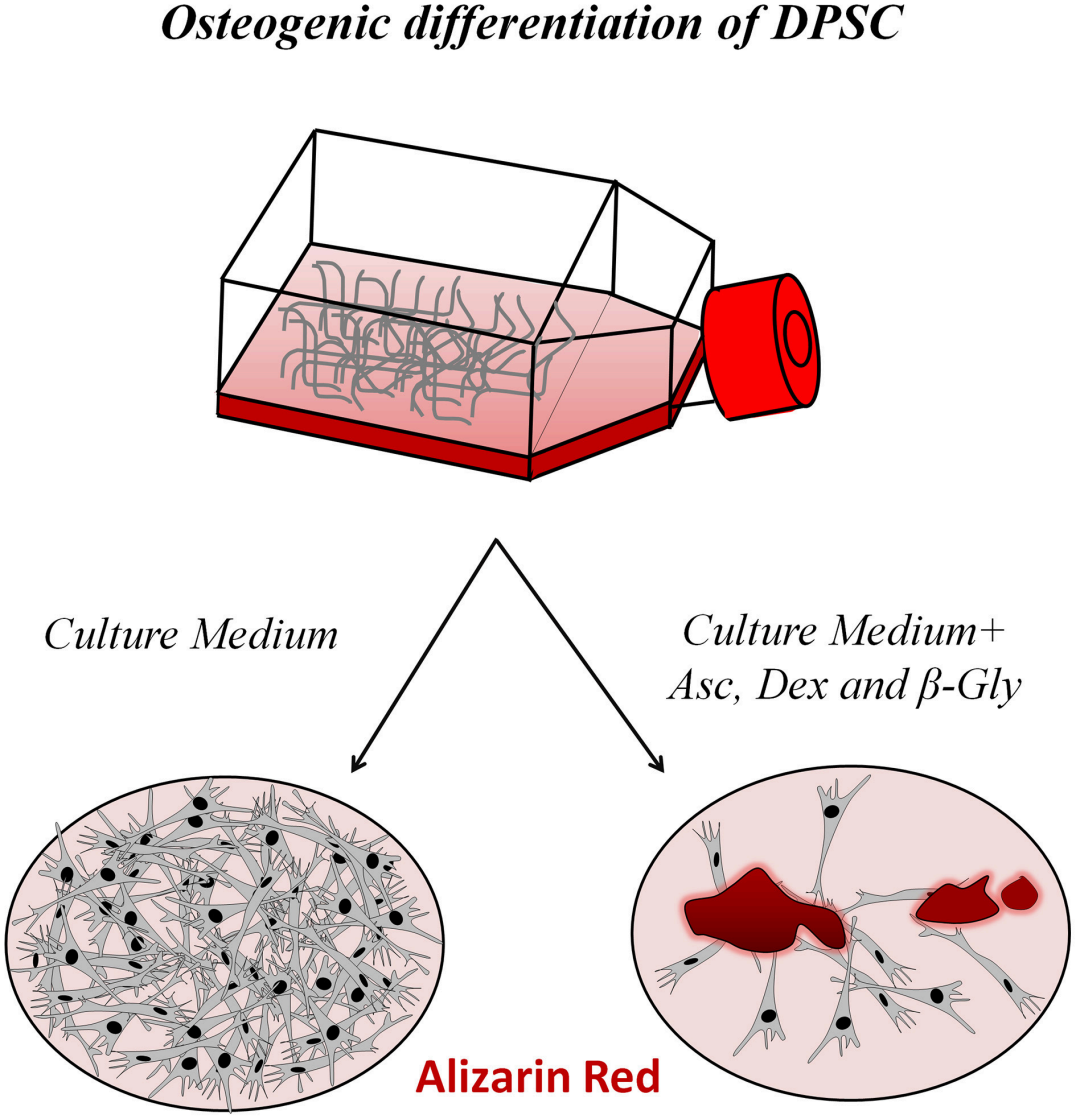
**Osteogenic differentiation of DPSC**. In the presence of a three-ingredient cocktail containing dexamethasone, β-glycerophosphate and ascorbate added to the culture medium for 7 days, DPSC differentiate to osteoblast-like cells that generate a mineralized bone tissue matrix that stains positively for Alizarin red.

The application of this differentiation cocktail is highly effective in inducing DPSC to generate a mineralized bone-like extracellular matrix *in vitro*. When DPSC are exposed to this differentiation cocktail for 7–21 days, they start to strongly express different osteoblast differentiation markers, such as Osterix, Runx2, osteocalcin, and bone sialoprotein. Alkaline phosphatase (ALP), and Alizarin red staining are positive under these conditions, and are used to demonstrate effective differentiation of DPSC to functional osteoblasts, and the generation of a calcified hydroxyapatite (HA)-containing extracellular matrix (Mangano et al., 2010; Yu et al., 2010; Li et al., 2011). DPSC-derived osteoblasts are phenotypically and functionally similar to normal primary osteoblasts, although some differences remain in terms of their gene expression profiles (Carinci et al., 2008). Naïve undifferentiated DPSC are negative for the adult osteo/odontoblast differentiation marker Osterix, but nevertheless exhibit detectable levels of Runx2, suggesting they are primed or pre-committed to differentiate to osteo/odontoblasts (Yu et al., 2010). When the osteogenic differentiation cocktail is added to DPSC, Runx2, and Osterix expression increase and these cells generate extracellular calcified bone-like matrix nodules, that stain distinctively and positively with Alizarin red (Laino et al., 2005; Figure 2).

In the last few years the use of DPSC for *in vivo* bone regeneration by tissue engineering has been extensively studied by relying on these protocols and using different experimental models and types of scaffolds with different results (Morad et al., 2013). Woven bone chips obtained by human DPSC (hDPSC) osteoinduction are able to induce the generation of adult bone tissue with an integral blood supply, when they are heterotopically transplanted in immune-compromised (IC) animals (d'Aquino et al., 2007). However, the use of scaffolding materials is often necessary to optimize the 3D structure of the formed bone tissue and enhance the osteoblastic differentiation of hDPSC. To this end, different materials have been successfully employed as a vehicle of hDPSC, including 3D gelatin scaffolds (Li et al., 2011), self-assembling biodegradable peptide nanofiber hydrogels (Chan et al., 2011), CaP/PLGA (calcium phosphate/polylactic-co-glycolic acid) scaffolds (Graziano et al., 2008a,b), particulate hydroxyapatite/tricalcium phosphate (HA/TCP) ceramic scaffolds (Zhang et al., 2008), and natural scaffolds like biocoral (Mangano et al., 2011) and chitosan/collagen (Yang et al., 2011). Finally, alginate and PuraMatrix™ hydrogels also provide a three dimensional (3D) biodegradable carrier of stem cells for bone tissue engineering purposes, with the additional large advantage that these formulations are injectable, forming 3D hydrogels upon contact with the physiological environment and thus completely filling the tissue lesion, with minimally invasive dental and orthopedic surgeries. Dental stem cells encapsulated in either alginate or PuraMatrix™ exhibited high capacities for osteo-differentiation both *in vitro* and *in vivo* (Misawa et al., 2006; Cavalcanti et al., 2013; Moshaverinia et al., 2013).

Inductive signals also play a critical role to stimulate development of new bone tissue. The development of a well-defined, safe, and controlled technique to obtain and locally deliver growth factors derived from autologous plasma has provided a powerful tool to enhance bone tissue regeneration, which has been successfully implemented in the clinical practice (Anitua et al., 2008, 2012). Formulations such as Platelet-rich plasma (PRP), Platelet-rich fibrin (PRF), and Plasma rich in growth factors (PRGF) provide a biologically enriched culture medium for the stimulation and functional differentiation of primary cells with latent regenerative potential. Several studies have shown that PRGF in particular provides additional benefits for bone regeneration (Paknejad et al., 2012; Rivera et al., 2013). It has been reported that PRP and PRGF induce proliferation and enhance osteogenic differentiation in SHEDs, DPSCs, and PDLSCs (Lee et al., 2011). Other studies have used recombinant human Bone morphogenetic protein 2 (rhBMP-2) to enhance osteogenic differentiation of DPSC in animal models of alveolar bone reconstruction (Liu et al., 2011). The use of rhBMP-2 is approved and widely extended to perform maxillary sinus floor augmentation in dental practice (Nazirkar et al., 2014; Freitas et al., 2015).

Despite a large body of preclinical evidence, very few clinical trials have still been performed to evaluate new advanced therapeutic medicinal products (ATMP) for bone regeneration based on DPSC (La Noce et al., 2014). A notable exception is one clinical trial performed with seven patients to repair mandibular bone defects by transplant of DPSC in a collagen scaffold (d'Aquino et al., 2009). After 3 years of follow-up, the clinical outcomes were positive (Giuliani et al., 2013). However, several obstacles remain, including a changing regulatory framework, a shortage of technology for automation of cell production by Good manufacturing practice (GMP) standards, a non-alignment between academic and industrial research, and a need for long-term product follow-up (La Noce et al., 2014; Leijten et al., 2015). These limitations have hampered the emergence of DPSC as a tool to enhance bone regeneration at clinical level.

## DPSC for dental pulp regeneration

Caries and root fracture are the cause of approximately fifty percent of all tooth extractions. Tooth injuries such as pulp exposure, deep caries and irreversible pulpitis are currently treated by endodontics and dental refilling/reconstruction. This procedure usually entails the amputation or entire removal of the dental pulp and replacement of the tissue with an inert material. Root fracture and tooth loss are closely related to this loss of pulp vitality because innervation and vasculature affect pulp homeostasis and root dentin regeneration. The regeneration of pulp injuries has become a goal for functional tooth restoration in dentistry (Nakashima and Akamine, 2005; Nakashima et al., 2009).

The first research reports on DPSC showed that these cells present optimal characteristics to attain functional regeneration of the dental-pulp chamber. Xenogenic transplant of HA/TCP scaffolds with hDPSC into the dermis of IC mice resulted in the formation of pulp-like tissue and polarized odontoblasts that produced tubular dentin, showing that dentinogenic differentiation was one of the prime or default programs established in the DPSC phenotype (Gronthos et al., 2000, 2002). Recently, more refined studies combining SHED with injectable scaffolds (Puramatrix™ and rhCollagen type I) have demonstrated that dental stem cells are not only kept alive when injected into human premolar root canals *in vivo*, but are also able to fully reconstruct vascularized pulp tissue throughout the root canal, and to differentiate to DSPP and DMP-expressing odontoblasts that generate new dentin (Rosa et al., 2013). In a similar experimental design, other authors employed combinations of DPSC and umbilical vein endothelial cells embedded in Puramatrix™ scaffold, to improve vascularization and angiogenesis of *de novo* formed dental pulp-like tissue (Dissanayaka et al., 2015).

Other studies have focused on the regenerative potential of different cytokines and growth factors, with the goal to improve cellular chemotaxis and cell homing into the emptied dental pulp space *in vivo*. Different combinations of cytokines and growth factors which included basic Fibroblast growth factor (bFGF), Platelet derived growth factor (PDGF), or Vascular endothelial growth factor (VEGF) in the presence of basal levels of Nerve growth factor (NGF) and BMP-7 were effective in regenerating a revascularized and recellularized dental pulp-like tissue integrated into endodontically-treated root canal dentinal walls *in vivo* (Kim et al., 2010). In addition, it has been shown that bFGF and Stromal-derived factor (SDF1) also exert a potent chemotactic recruitment on DPSC, while BMP-7 induces their differentiation (Suzuki et al., 2011). In a recent study, SDF1 loaded onto silk-fibroin scaffolds promoted the complete regeneration and revascularization of the dental pulp of mature dog teeth that had been subjected to endodontic treatment *in situ* (Yang et al., 2015), suggesting a promising future for the functional reconstruction of dental pulp tissue in next generation dentistry.

## DPSC for complete dental regeneration

In 2009, a ground-breaking report was published by the group of T. Tsuji on the generation of complete and fully functional teeth by combining isolated mouse dental epithelial and MSC (Ikeda et al., 2009). More recently, in 2011, the same research group developed a complete tooth unit consisting of a mature tooth, periodontal ligament and alveolar bone, using a similar recombination technique (Oshima et al., 2011). These tooth units were transplanted into toothless mouse jaw regions *in vivo*, and erupted correctly and in occlusion. They also presented an adequate dental structure, an adequate hardness of enamel and dentin, a proper function of the periodontal ligament, a positive alveolar bone remodeling response to orthodontic forces, and a positive response to noxious stimuli such as mechanical stress and pain.

Although precise replication of these results has not yet been reported on the literature, the aforementioned work represents a substantial advance in stem cell combination technology, with the potential to develop completely new bioengineered replacement organs for next generation regenerative therapy. One of the main limitations to date is probably the lack of consistent sources of epithelial cells that can be combined with dental mesenchymal cells (DPSC or others) to generate a complete bioengineered tooth germ. The team of T. Tsuji employed embryonic mouse tooth germ epithelia as precursors of the enamel organ, a structure that fully disappears in adult teeth. In the search for dental-competent sources of epithelial stem cells, some authors have reported success using adult gingival epithelial cells, which upon recombination with mesenchymal cells generate mature teeth and form enamel and dentin (Angelova Volponi et al., 2013). Other authors have employed iPS-derived cells to generate mature bioengineered teeth by similar recombination methods (Wen et al., 2012; Cai et al., 2013; Otsu et al., 2014). The substantial progress made in the field of dental bioengineering warrants further research on this line for the next years.

## DPSC for regeneration of nerve tissue and damaged cranial nerves

DSPC originate from the neural crest (Janebodin et al., 2011) and share a common origin with peripheral nerve glial progenitor cells (Kaukua et al., 2014). These features make DPSC a very interesting choice for regeneration of the peripheral nervous system, including nerves of the oral cavity. Some reports claim that DPSC can even differentiate to functionally active adult neurons (Arthur et al., 2008; Kiraly et al., 2009; Gervois et al., 2015). These conclusions are based on the acquisition of neuron-specific gene and protein markers by DPSC, and also on their capacity to generate neuronal-like voltage-dependent sodium and calcium currents, and action potential-like membrane voltage oscillations. However, it should be noted that to date there has been no definite proof of differentiation of DPSC to genuine neurons that can exhibit repetitive firing of action potentials upon electrical stimulation, and establish synapses showing functional plasticity as identified by transmission electron microscopy (TEM).

**Figure 3:**
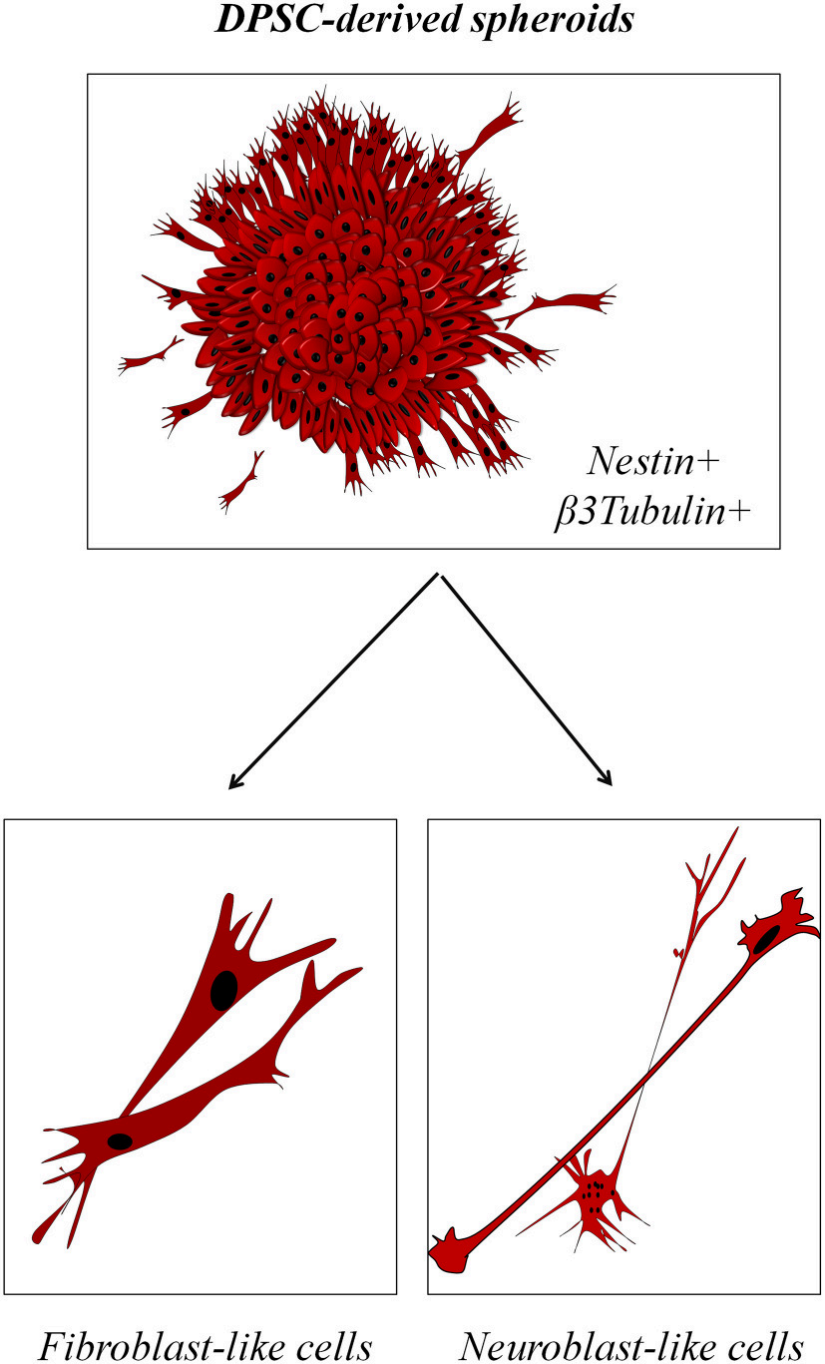
**Neurosphere-like properties of DPSC-derived spheroids**. **(Top)** DPSC grown in serum-devoid conditions rearrange to form characteristic spheroids that stain positive for neural stem cell markers. **(Bottom)** Migratory cells outside of the spheroids express some neuronal markers and present a variable morphology, with either fibroblast-like or neuroblast-like features.

However, it is undeniable that DPSC present some striking similarities with neural stem cells. When DPSC are grown in culture conditions lacking fetal serum, they reorganize morphologically and switch from a uniform cell monolayer to a more quiescent state characterized by the appearance of prominent spheroid structures that resemble CNS-derived neurospheres which stain positively for the neural stem cell marker Nestin (Ibarretxe et al., 2012; Bonnamain et al., 2013; Xiao and Tsutsui, 2013; Gervois et al., 2015; Figure 3). Migratory cells are occasionally observed to leave the DPSC spheroids and virtually all these cells express the neuron-lineage marker β3-tubulin. Moreover, their morphology is in some cases surprisingly similar to migratory neuroblasts, with a long and thin leading process terminated by a prominent growth cone, and a trailing cell body displaying a few short cytoplasmic processes (Marín et al., 2006). However, a note of caution should be added: another cell type of the dental pulp, the odontoblast, is also characterized by the expression of Nestin, the generation of voltage-dependent Na+ currents, and a similar morphology (Fujita et al., 2006; Ichikawa et al., 2012). The DPSC population grown in these conditions is fairly heterogeneous, both morphologically and physiologically, with a range of different morphologies from fibroblast-like to neuroblast-like cells, and also a variable rate of response to stimulation with neurotransmitter receptor agonists.

Regardless of the debate on the true capacity of DPSC to differentiate to neurons and other neural cell lineages, there is little doubt that these cells could be a very interesting choice to repair injured nerve tissues as a result of their active secretion of neurotrophic and immune-modulatory factors (Nosrat et al., 2004; Pierdomenico et al., 2005). The high expression of certain neural markers and neurotransmitter receptors by DPSC also suggests that these cells may actively respond to signals of the neural environment and effectively integrate into injured nerve tissues, promoting the reestablishment of functional nerve connectivity (Arthur et al., 2009; Kadar et al., 2009; Kiraly et al., 2009). For instance, some recent reports show that transplanted DPSC are able to differentiate *in vivo* to myelinating cells in the spinal cord, replacing lost cells and promoting regeneration of transected axons in acute models of spinal cord injury (Sakai et al., 2012).

In the case of the peripheral nerve system, treatment of nerve injuries represents a clinical challenge because of the difficulties of regenerating transected nerves. Though numerous surgical successes have been reported with a short nerve gap, there is still no satisfactory technique for long nerve defects, which often require a complex clinical reconstruction. Autologous nerve grafting has been considered the gold standard for repairing peripheral nerve gaps caused by traffic accidents or tumor resectioning (Kumar and Hassan, 2002; Hayashi and Maruyama, 2005; Bae et al., 2006). However, this technique has inevitable disadvantages, such as a limited supply of available nerve grafts and permanent loss of the sacrificed donor nerve function. Brain-derived neural progenitor cells also promote regeneration of transected nerves (Murakami et al., 2003). However, the use of cells from other neural tissues involves potentially serious clinical complications along with ethical considerations.

Taking all these arguments into account, there is an active search for new sources of cells to be used in craniofacial nerve bridging and regeneration. Considerable advances have been made in this field using DPSC for the treatment of facial nerve injuries. Particularly, patients with facial paralysis, especially younger ones, may experience tremendous psychosocial distress about their condition (Chan and Byrne, 2011). Recent studies have used DPSC transplanted in PLGA tube scaffolds to achieve a complete functional regeneration of the facial nerve in rats to recovery levels comparable to those obtained with peripheral nerve autografts (Sasaki et al., 2011, 2014). Interestingly, recent research also indicates that hDPSC can be differentiated to Schwann-like cells that efficiently myelinate DRG neuron axons *in vitro*, a finding confirmed by ultrastructural TEM analysis (Martens et al., 2014). Considering the important role that Schwann cells play in axonal protection and regeneration of peripheral nerves (Walsh and Midha, 2009), and the difficulty of their harvesting and maintenance, the generation of DPSC-derived autologous Schwann cells may represent a milestone in the design of new treatments for conditions of peripheral nerve injury, including facial paralysis.

Finally, another important property of DPSC is their active secretion of neurotrophic factors (Nosrat et al., 2004; Bray et al., 2014), which may be exploited to treat neuropathic pain states associated with peripheral nerve injury. In the case of orofacial pain, some of the most distressing and painful conditions that can be experienced by a human being are neuralgias affecting the trigeminal nerve, or CN V. These are characterized by intense stabbing pain and spasms, usually associated with a mechanical injury, compression, demyelination and inflammation of trigeminal sensory fibers (Love and Coakham, 2001; Sabalys et al., 2013). It is known that local application of Glial derived neurotrophic factor (GDNF) exerts a potent analgesic effect and reverses the symptoms associated with neuropathic pain (Boucher et al., 2000). Because DPSC secrete important amounts of GDNF, it is conceivable that autologous DPSC transplantation to the injured trigeminal nerve may also potentially provide important benefits to treat severe orofacial pain disorders, alone or in combination with pharmacological enhancers of GDNF signaling (Hedstrom et al., 2014), some of which are even formulated to permit topical application.

## DPSC for salivary gland regeneration

Salivary gland regeneration is an area of intense research because the most common treatment for head and neck cancer (i.e., radiotherapy) irreversibly affects salivary gland function, causing hyposalivation and xerostomia. In consequence, difficulty in mastication, swallowing, taste, speech, rampant dental caries and mucosa infections appear, leading to high discomfort in patients and a reduced quality of life (Vissink et al., 2010). Palliative treatments such as mechanical stimulation of salivary gland activity (chewing gum), or pharmacological agents such as pilocarpine (Salagen™), as well as saliva substitutes, show a limited efficacy in relieving the symptoms (Taylor, 2003).

Tissue engineering of a salivary gland replacement organ would be a conclusive way to treat these patients. To accomplish this complex task, there are some issues that must be taken into account: (i) optimization of the 3D scaffold material properties (including non-toxicity, permeability and biodegradability, among others), (ii) identification of an epithelial stem cell population capable of appropriate salivary differentiation (iii) definition of ideal culture conditions and extracellular matrix components (ECM) with laminin, integrins, MMPs to mimic the microenvironment of the gland, and (iv) selection of a population of MSC to be combined with salivary epithelial cells to generate a full bioengineered salivary gland germ. Finally, in comparison with dental bioengineering, salivary gland bioengineering presents an important additional requirement: apart from the communication between the epithelium and the mesenchyme, epithelial-axonal interactions are also required from the very beginning of salivary gland morphogenesis, because innervation plays an important role in regulating the activity of epithelial progenitor cells (Knox et al., 2010) and salivary gland branching and tubulogenesis (Nedvetsky et al., 2014). Therefore, salivary gland bioengineering strategies must also take into account that this is an organ that heavily relies on the establishment of a functional innervation to properly complete its development (Lombaert et al., 2011; Knosp et al., 2012).

Some studies indicate that isolated mouse salivary gland stem cells (cKit+, CK14+, Msi-1+, and Sca-1+) can grow and form salispheres that produce amylase (Lombaert et al., 2008). Moreover, injection of a liquid suspension containing only 300–400 cKit+ cells into the mouse SMG was shown to partially rescue the loss of morphology and function induced by irradiation insults (Lombaert et al., 2008; Coppes and Stokman, 2011; Nanduri et al., 2013). In recent years, the technology to generate salivary gland organoids *in vitro* has progressed remarkably. Recent major advances in the field include the advent of bioengineered glands constructed from dissociated cells from adult and fetal salivary glands (Ogawa et al., 2013; Nanduri et al., 2014). An important study by the group of T. Tsuji showed that the bioengineered salivary glands generated by a cell recombination protocol contained epithelium, mesenchyme, endothelium, and nerve cells. Most importantly, the entire organ could be transplanted into adult mice, reconnect with the existing ductal system and function properly (Ogawa et al., 2013; Ogawa and Tsuji, 2015). Thus, the salivary gland joins the tooth in the list of organs that can be fully generated *ex vivo* by tissue engineering. As in the case of the tooth, the main challenge to translate these findings to clinical practice is still the need for sources of stem cells that are sufficiently abundant and competent for salivary epithelial differentiation, and that do not involve destruction of donor salivary gland tissue.

In the search for stem cell sources to be employed in salivary gland bioengineering, some progress has been recently made using DPSC. It has been shown that DPSC contribute to the generation of salisphere-like structures when combined with Human salivary gland (HSG) cell lines in either Matrigel™ or hyaluronic acid hydrogel scaffolds (Janebodin and Reyes, 2012). These salispheres expressed markers of terminally differentiated acinar cells, and generated a well-irrigated salivary gland-like tissue after subcutaneous transplantation in IC mice. The principal role of DPSC in salivary gland bioengineering is to generate the salivary stroma or mesenchymal-derived compartment, and these cells are in theory one of the best choices for that purpose because the tooth mesenchyme and the salivary gland mesenchyme share a common embryonic origin (i.e., neural crest).

**Figure 4:**
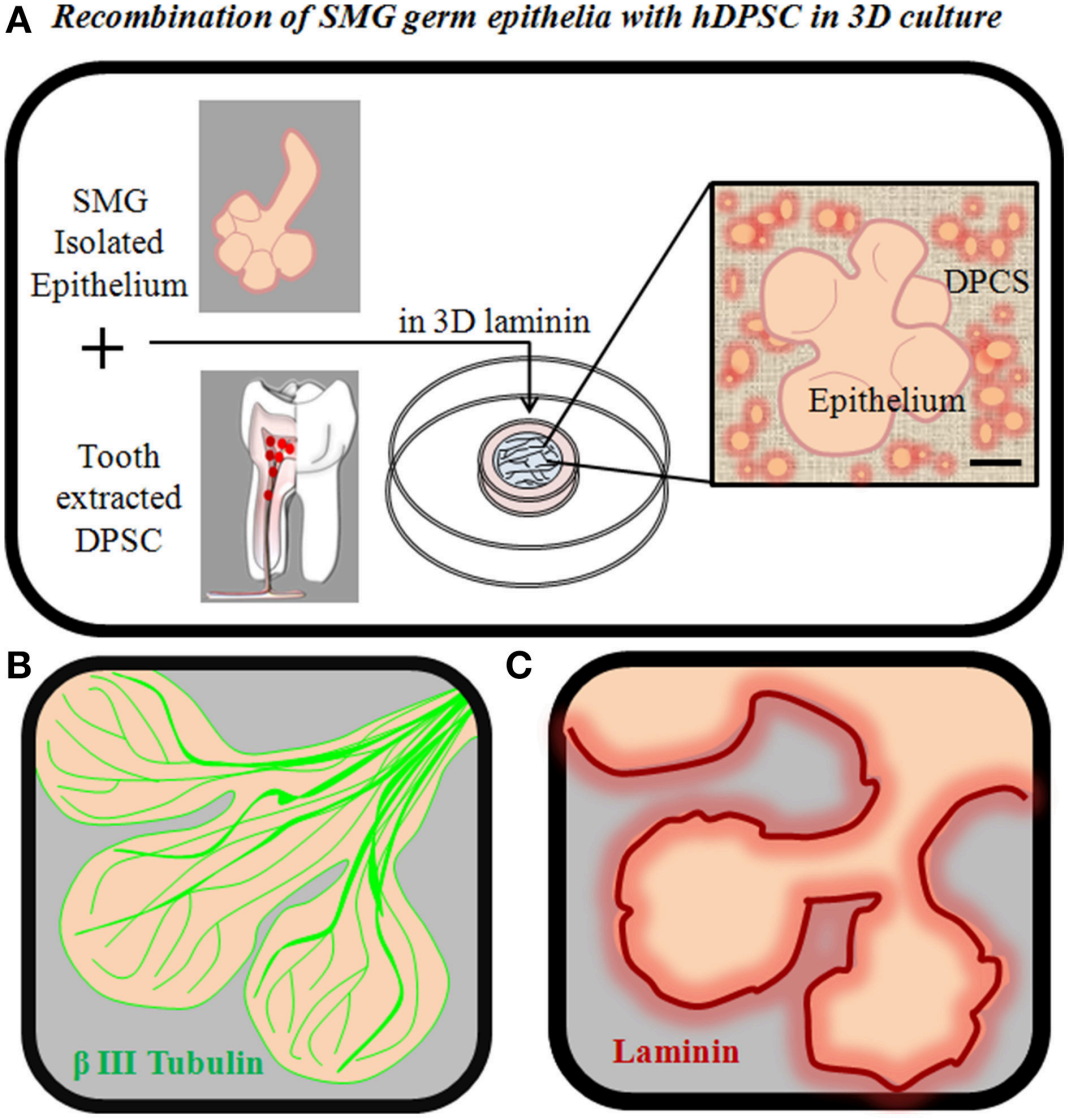
**Recombination of salivary gland germ epithelia with DPSC in 3D culture. (A)** SMG epithelia can be mechanically and enzymatically isolated from SMG mesenchyme, and recombined with hDPSC in 3D laminin scaffolds to generate a bioengineered salivary gland germ **(B)** SMG acini from E14.5 mouse, showing the extensive natural innervation of the SMG by β3-tubulin+ nerve fibers at this developmental stage. **(C)** SMG acini from E14.5 mouse, showing the natural localization of Laminin in the basal lamina that separates the salivary epithelium and mesenchyme.

HSG are stable cell lines derived from neoplastic tissues (Shirasuna et al., 1981) and definitely not the best choice to bioengineer a physiologically functional salivary gland. In order to evaluate whether trophic support provided by DPSC sustains proliferation and ramification of primary salivary gland epithelia in developmental stages, *in vitro* recombination experiments between isolated embryonic mouse salivary epithelia and dissociated hDPSC may be performed in 3D laminin or other extracellular matrix scaffolds (Sequeira et al., 2013). In these conditions, DPSC bind and interact with isolated salivary epithelia, improving the *in vitro* viability of the tissue (Figure 4). However, recombination with DPSC alone is clearly insufficient to promote long-term growth and ramification of developing salivary epithelia. One missing but important potential factor is that this system cannot reproduce the extensive innervation that occurs within normal salivary gland tissues and plays a crucial role in salivary gland growth and morphogenesis (Knox et al., 2010; Knosp et al., 2012). Next steps in this direction should involve the addition of specific growth factors that sustain epithelial cell growth and commitment to salivary gland cell differentiation, as well as the use of coculture systems between bioengineered salivary glands and nerve cells to attempt to mimic the growth stimulatory effect of natural salivary innervation.

## Concluding remarks

DPSC have emerged as a very promising tool with a great potential to be used in tissue engineering models aimed at the functional reconstruction of different craniomaxillofacial organs. Some of the main advantages of these cells are their multifaceted differentiation capacity, along with their non-tumorigenic phenotype, a high proliferation rate, a relative simplicity of extraction and culture, and their possibility of cryopreservation, which ultimately make possible to obtain patient-specific cell lines for their use in autologous cell therapy. However, many issues and challenges still need to be addressed before these cells can be employed in clinical practice. The full control of differentiation of DPSC to specific fates is still one important issue, and although DPSC derivation to certain connective tissue-lineage cells appears to be relatively simple, their differentiation into nerve-tissue lineages still poses important unanswered questions. Moreover, the development of the cell recombination technology required to create next generation bioengineered replacement organs will necessitate extensive research for the coming years. In this case, DPSC may be an ideal choice to be used in experimental models of tissue-engineered reconstruction of organs of the oral cavity, and the paradigmatic cases of the tooth and the salivary gland may constitute the leading edge of a massive research effort on next generation organ replacement therapies.

## Funding

This work was financed with project grants from the University of the Basque Country UPV/EHU (UFI11/44) and the Basque Government (IT831-13). MA, PG, and JL were sponsored by the University of the Basque Country (UPV/EHU) and VU by Jesus Gangoiti Barrera Foundation.

### Conflict of interest statement

The authors declare that the research was conducted in the absence of any commercial or financial relationships that could be construed as a potential conflict of interest.
